# Endoscopic Transsphenoidal Sellar Surgery via One Nostril: Own Experience and Systematic Review of the Literature

**DOI:** 10.3390/life15081233

**Published:** 2025-08-04

**Authors:** Stefan Linsler, Bernardo Reyes Medina, Safwan Saffour

**Affiliations:** Klinik für Neurochirurgie, Klinikum Bayreuth, Medizincampus Oberfranken FAU Erlangen, 95455 Bayreuth, Germany; bernardo.reyesmedina@klinikum-bayreuth.de (B.R.M.); safwan.saffour@klinikum-bayreuth.de (S.S.)

**Keywords:** endonasal, transsphenoidal, endoscopy, sellar surgery, mononostril, binostril, meta-analysis

## Abstract

**Background:** Endonasal endoscopic approaches to the skull base are still under investigation, with research aiming to achieve minimally invasive procedures that maximize resection while minimizing complications. This study shares our experience with a mononostril technique and compares it with the existing literature on mononostril approaches for sellar lesions. **Methods:** A systematic review of eight large series, totaling 1520 patients who underwent endoscopic mononostril transsphenoidal surgery, was performed. The surgical technique was detailed, and parameters such as resection completeness, operative time, complications, and nasal symptoms were analyzed. **Results:** Gross total resection ranged from 56% to 100% for non-functioning adenomas, 54% to 89% for hormone-secreting adenomas, and 83% to 100% for other sellar lesions. The most common complications were CSF leaks (1.5–4.1%) and nasal issues, such as epistaxis or sinusitis (0–6%). Internal carotid artery injury occurred in 0–1% of cases. The average surgical duration was 87 to 168 min. **Conclusions:** The mononostril approach offers comparable resection rates, CSF leak risks, and morbidity to binostril or microsurgical methods. The mononostril approach is fast, minimally invasive, and preserves the nasal mucosa, making it a viable option for many sellar lesions.

## 1. Introduction

The treatment of sellar lesions has evolved significantly, particularly with advancements in neurosurgical techniques over recent decades [1]. The microsurgical transsphenoidal approach has been investigated and refined over nearly a century of research. This method has long been the gold standard due to its ability to achieve precise resection with low morbidity, leading to favorable patient outcomes [2,3,4,5,6,7,8,9,10,11]. Moreover, the microsurgical transsphenoidal approach has proven effective in safely removing sellar tumors with good outcomes.

Since the early 1990s, intracranial endoscopy has rapidly evolved [12,13,14,15], increasing interest in endoscopic techniques for skull base surgery. Numerous studies highlight the less invasive nature of endonasal endoscopic approaches [16,17,18,19,20,21,22], demonstrating feasibility even for lesions approaching the clivus [23] and brainstem [24].

Studies have increasingly documented the advantages of endonasal endoscopic approaches, which are less invasive and have proven feasible even for complex lesions near the clivus and brainstem. As surgeons gained experience, they successfully managed increasingly complex pathologies. Nonetheless, the optimal endoscopic technique for sellar lesions remains debated [25,26,27,28,29].

In the late 1990s, some groups pioneered endoscopic mononostril transsphenoidal procedures for pituitary adenomas, developing their own techniques [16,18,30,31,32,33]. Today, most neurosurgeons favor a binostril approach for skull base lesions [34,35,36,37], though some still prefer the mononostril method for sellar tumors [19,38,39,40,41,42,43]. Recent studies have focused on better results and the feasibility of resecting pathologies in the cavernous sinus and around the internal carotid artery [44].

In this study, we present our mononostril endoscopic technique for treating sellar lesions. We compare our results with other reported series, conduct a meta-analysis, and discuss the distinctions between the binostril and mononostril approaches. This comprehensive examination aims to clarify the efficacy and applicability of the mononostril technique in the context of contemporary practices in neurosurgery.

## 2. Materials and Methods

### 2.1. Patients and Surgical Technique

Over the last decade, the authors have treated patients with pituitary adenomas and various other sellar and parasellar lesions using an endoscopic endonasal transsphenoidal approach, with detailed follow-up evaluation data. All patients received preoperative evaluations for endocrine function and visual capability, which included formal testing of their visual fields. Postoperative assessments were carried out during the initial week of hospitalization and again six weeks after surgery, with further follow-up based on hormonal and MRI results. Standard pre- and postoperative MRI scans were conducted, along with CT scans featuring axial and coronal reconstructions to delineate the bony structures of the sellar area, including the sphenoid cavity. Details of this patient cohort and initial results with the mononostril technique are described extensively in recent publications [19,20,21,22,40,42,45,46].

In this review, the authors compared their patient cohort with other published series of mononostril approaches to the sellar region and conducted a detailed meta-analysis following the PRISMA 2020 guidelines [47,48]. See also Supplementary Materials.

### 2.2. Review of the Literature

A systematic literature review was conducted following PRISMA guidelines to enhance understanding of the topic and provide valuable insights to the medical community. Our research involved searching PubMed, Web of Science, and Scopus databases. The search terms included “endoscopy,” “endonasal,” “one nostril,” “mononostril,” “transsphenoidal surgery,” “pituitary adenoma,” and “sellar pathologies.” All references within these articles were also reviewed. Additionally, we identified articles from the references of the retrieved publications. The date of the search was April 2025.

#### 2.2.1. Inclusion and Exclusion Criteria

All English language studies that addressed mononostril approaches were included. Non-English language studies, no full-text available studies, case series, and case reports were excluded from our analysis.

#### 2.2.2. Study Selection, Data Extraction, and Quality Assessment

Two researchers, S.L. and B.R., independently reviewed study titles and abstracts based on predefined search criteria to identify studies that met the inclusion requirements. The same researchers then evaluated the full texts for inclusion and gathered relevant data. In instances of disagreement, a third reviewer, S.S., was consulted. Following this, B.R. and S.L. analyzed all selected articles and compiled the data using SPSS (version 22.0, IBM Corporation, Armonk, NY, USA). For each included article of this meta-analysis, typical study characteristics such as study design, year, country, sample size, and database used were extracted, along with all reported parameters. These parameters included surgical technique, radicality, complications, pathology, and details of surgical method. Patient-related factors and predictors included age, sex, and surgical approach. Not a single study was a randomized clinical trial.

**Figure 1:**
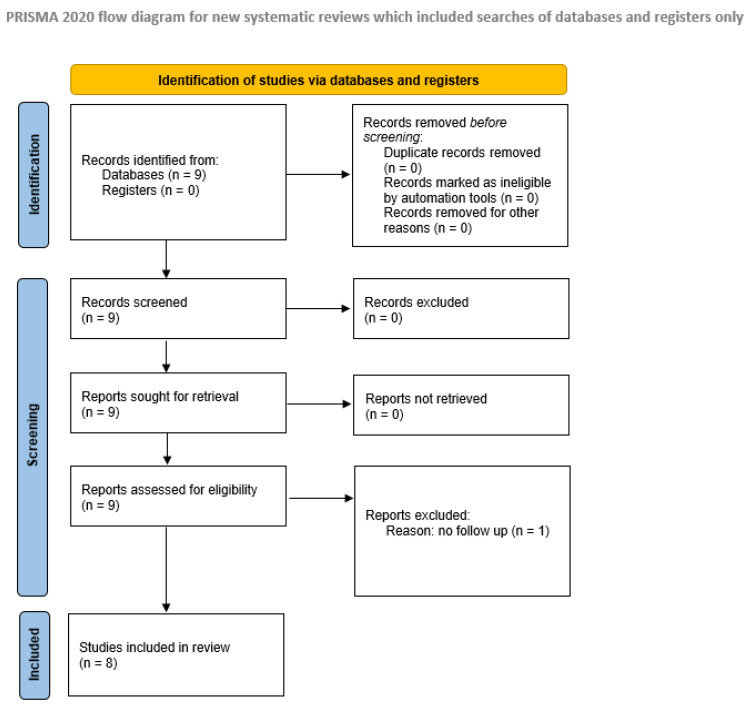
Flowchart illustrating the process of the identification of the included studies in accordance with PRISMA guidelines 2020. In total, data from 1520 mononostril transsphenoidal procedures across these eight series were analyzed. Details on cohort composition and follow-up evaluations are summarized in Table 1.

The search yielded nine series on endoscopic mononostril techniques by Jho (2001), Cappabianca et al. (2002), Linsler et al. (2013), Han et al. (2013), Eseonu et al. (2018), Darwish et al. (2018), Oertel et al. (2020), Peeters et al. (2021), and Baussart et al. (2021) [16,19,33,38,39,41,42,43,49]. Baussart et al. described their surgical technique in detail and reported over 2000 cases; however, due to the lack of follow-up evaluation clinical data, their study was excluded from the meta-analysis. The details of the literature search are provided in Figure 1.

### 2.3. Statistics

The illustration and analysis of data were performed using SPSS (version 22.0, IBM Corporation, Armonk, NY, USA) and Excel (Microsoft Corp., version 2003, Redmond, DC, USA). The patient cohorts were compared using the Mann–Whitney U Test. Fisher’s exact test was applied to compare differences between values in the groups. Additionally, uni- and multivariate analyses were performed. The significance level was set at *p* < 0.05.

## 3. Results

### 3.1. Pathologies, Radicality, and Surgical Time

The histological diagnoses and mean age are demonstrated in Table 1 for all series. There were more female than male patients in the series. A total of 38–68% of all pathologies were non-functioning adenomas. Most frequent secreting adenomas were GH-secreting adenomas (0–25%), prolactinomas (4–34%), and ACTH-secreting adenomas (2.5–26%). Additionally, meningiomas 0–11%, craniopharyngiomas 0–9%, clival chordomas 0–7%, and Rathke’s cleft cyst in 0–6% of cases were treated.

The radicality ranged from 56% to 100% for non-functioning adenomas, from 64% to 89% for hormone-secreting adenomas, and from 83 to 100% in all other sellar pathologies. The recurrence rate ranged from 2 to 8.2%. The details are presented in Table 2.

### 3.2. Complications

The most common complications were CSF leakage, occurring in 1.5–4.1% of cases, and nasal issues such as epistaxis and sinusitis, which were reported in 0–6% of cases. Worsening of visual function was observed in 0–5.6% of cases, while diabetes insipidus ranged from 0.4 to 5%, and new postoperative hormonal deficits affected 3–9.3%. Internal carotid artery (ICA) injuries were reported in 0–1% of cases. Overall, the complication rate across all mononostril transsphenoidal procedures was very low. However, there were two deaths (mortality rate of 0.1%), which were most likely due to ICA injury. All complications are summarized in Table 3.

In our series, we also analyzed nasal complaints postoperatively: five patients reported nasal congestion or reduced airflow, none of which were severe. One patient was found to have a septal perforation during follow-up evaluation, which was likely responsible for persistent nasal complaints. Three additional patients required revision due to nasal mucosal adhesion. In other series, sinusitis was reported in 0–6% of cases, and nasal discomfort or cacosmia in 0.4–2.1%. No further sinonasal complications were detailed.

### 3.3. Details of the Different Mononostril Surgical Techniques

Following disinfection and vasoconstriction, the procedure commences with an endoscopic examination of the nasal cavity through one nostril. Given the confined space, the endoscope and instruments are meticulously introduced under direct endoscopic visualization until they reach the middle turbinate. The speculum is then gently advanced along the inferomedial side of the middle turbinate, often assisted by lateral fluoroscopy, to access the sellar floor and sphenoid sinus. Fluoroscopy in combination with neuronavigation is particularly helpful for intraoperative orientation, especially during the learning curve, and was used in 62% of cases across five of eight series. The nose is gently dilated in multiple steps, preserving the nasal mucosa and reducing bleeding. Some authors, such as Eseonu, Baussart, and Darwish, describe their three-hand technique without using a speculum, while others, such as Peeters et al., alternate between a three-hand and a two-hand technique with a speculum. In most cases, after inserting the speculum, the endoscope is fixed in an endoscope holder, although some groups operate with a free hand. The septal mucosa is coagulated, incised, and mobilized laterally with a microdissector to expose the sphenoid ostium, located at the recessus spheno-ethmoideus. The mucosa is then removed or pushed aside as necessary. The septum is broken at the sphenoid floor by pushing the speculum against it, allowing entry into the sphenoid sinus. The speculum remains in place, usually at the sphenoid floor, providing an unobstructed view behind the septum. The sphenoid ostium is identified, and the sinus is entered using Kerrison rongeurs. The sphenoid floor is carefully removed with a small rongeur, preserving a large piece for later reconstruction. If the sphenoid mucosa is prominent, it is removed or pushed aside after mobilization. Intraoperatively, landmarks such as the sphenoethmoid planum, clivus, and the bony prominences of the carotid arteries and optic nerves are identified. Once adequate exposure is achieved, the sellar floor is opened, and the dura is incised. The lesion is then removed in a piecemeal fashion using curettes, suction, and forceps. For tumors with suprasellar extension, a Valsalva maneuver may help bring residual tissue and the diaphragm into view. At the end of the procedure, the 0° scope is replaced with angled scopes (30° or 45°) to visualize the lateral borders of the sella, carotid arteries, and surrounding structures. Thus, a high radicality can be achieved with angled endoscopes [46]. In addition, the healthy pituitary tissue can be identified at the end of the procedure in most cases, as previously reported in detail [20,21]. The details are presented in Table 4.

The nose is gently dilated in multiple steps, preserving the nasal mucosa and reducing bleeding. Some authors, such as Eseonu, Baussart, and Darwish, describe their three-hand technique without using a speculum, while others, such as Peeters et al., alternate between a three-hand and a two-hand technique with a speculum. In most cases, after inserting the speculum, the endoscope is fixed in an endoscope holder, although some groups operate with a free hand. After tumor removal, if the diaphragm is thin or a CSF leak occurs, the sella is closed using an autologous periumbilical fat graft and fibrin glue. In cases with an intact, stable diaphragm, the sella is simply filled with fibrillar hemostatic agents (e.g., Tabotamp fibrillar, Surgical fibrillar, and Duragene). The sellar floor is reconstructed with bone pieces and, if required, covered with fibrin glue or Tachosil. Some authors also used a vascularized nasoseptal flap for closure (*n* = 2). The nasal septum is repositioned to its midline using a finger or dissector inserted deeply into the opposite nostril. A final inspection is performed with a 0° scope in a freehand technique. Nasal packing is applied if adequate hemostasis cannot be achieved elsewhere to prevent mucosal synechiae. Retrospective analysis showed that nasal tamponade deployment was associated with reduced sinusitis (*p* = 0.002), fewer olfactory disturbances (*p* = 0.031), and improved nasal breathing (*p* = 0.026) [50]. Perioperative complications, surgical duration, nostril selection, pathology type, extent of resection, and surgical intent showed no significant impact on sinonasal outcomes. Therefore, the authors recommend the routine use of nasal tamponades to enhance sinonasal recovery after endoscopic endonasal procedures.

Additionally, analysis revealed no statistically significant differences in complication rates, completeness of resection, or recurrence rates between surgical methods and the use of a speculum or endoscope holding device (*p* > 0.1).

## 4. Discussion

Over almost a century, one of the main topics in neurosurgery has been the search for minimally invasive techniques to minimize potential damage to adjacent structures [1]. This is especially in focus in modern skull base surgery. Currently, surgical strategies for endonasal approaches to the anterior skull base and sellar region are evolving in different directions. Advances in intracranial endoscopy and HD scopes have increased the relevance of endoscopic techniques in skull base surgery. The benefit of endoscopic techniques for endonasal skull base surgery compared with microscopic techniques has been reported several times [51,52,53,54,55,56]. Many neurosurgeons have worked to make the transsphenoidal approach more minimally invasive. Pioneering efforts by Jho and others on endonasal endoscopic resection have been applied to various skull base lesions, including pituitary adenomas and anterior cranial fossa meningiomas. Jho employed a one-nostril approach without the use of a speculum. He published a series of reports on this technique over several years [18,32,33]. Cappabianca and co-workers also used a very similar technique without a speculum through one nostril. They thought that the speculum creates a rigid tunnel that limits visibility and maneuvering of the instruments [16]. Furthermore, they stated that the endoscope—avoiding the use of the nasal speculum—enables a widening of the working angle in all directions and greater angles of view for inspection [16].

The authors of this meta-analysis also evaluated their own technique. After several modifications, the described surgical approach was used for intra- or perisellar lesions when a transsphenoidal route was considered feasible. Jho and co-workers [18,32,33], Cappabianca [16,17,57] and colleagues, Eseonu and colleagues [38], Darwish et al. [43] and Baussart and colleagues [49] did not use a speculum in their techniques. In the authors’ experiences, repeatedly inserting and removing the endoscope and instruments during surgery can potentially injure the nasal mucosa. It is important to note that the instrument is not controlled by the endoscope until it appears in front of it. Using a speculum allows the endoscope to be fixed in front of the sphenoid sinus—and often in the sella—enabling instrument movement in and out without mucosal contact. Most studies on endoscopic endonasal techniques for sellar lesions focus on binostril approaches, which provide excellent visualization and facilitate instrument handling within the relatively narrow surgical corridor [23,36,37,58,59]. Based on the reported surgical results, skull base surgery has evolved significantly: currently, manipulation of the cavernous sinus compartments is more systematic, and in some cases, neurosurgeons must mobilize or work close to the intracavernous carotid artery. This demands optimal instrument maneuverability and wide angles of approach, which is why many centers prefer the binostril or extended endonasal approaches for these indications. Based on the reported surgical results, the skull base surgery has evolved significantly: nowadays, manipulation of the cavernous sinus compartments is more systematic, and in some cases neurosurgeons must mobilize or work close to the intracavernous carotid artery. This demands optimal instrument maneuverability and wide angles of approach, which is why many centers prefer the binostril or extended endonasal approaches for these indications. The authors are in line with these reports and propose a binostril approach in these special cases also.

Han et al. compared in their series a total of 250 patients, of which 200 underwent a mononostril approach and 50 underwent a binostril approach. They demonstrated a better result for surgical time, blood loss, and nasal complications for the mononostril group with comparable radicality [39] (details see Table 5). Conrad et al. published a small series of binostril and mononostril surgeries with additional anatomical analyses. The authors concluded in their manuscript that binostril endoscopic methods are preferable for treating larger tumors with notable parasellar and suprasellar extension, as well as in extended approaches, due to the improved maneuverability of instruments. Conversely, for smaller tumors, mononostril approaches combined with a nasal speculum are not only significantly faster but also effective [25] (details see Table 5). The authors of this review have the same experience: 65 patients were treated via the binostril or mononostril endoscopic endonasal extended approach as previously published [25,42]: The binostril approach was significantly more time-consuming than the mononostril technique. It provided a superior panoramic view due to a wider opening of the sella in both craniocaudal and horizontal directions. However, the need to remove more of the nasal septum was a disadvantage. The authors concluded that the binostril approach is superior for large tumors with parasellar extension and those requiring extended procedures. Conversely, the mononostril technique is preferable for tumors with limited intra- and suprasellar extension [25]. Linsler et al. published an anatomical study focusing on the extent of dorsal septectomy via both mono- and binostril approaches to the sellar region. They were able to visualize all key landmarks of the sellar, suprasellar, and parasellar regions through a single nostril in nearly every cadaver, without performing septectomy or turbinectomy. Based on these findings, they concluded that the mononostril approach is less traumatic to the nasal cavity [27]. There is only one meta-analysis in the literature comparing binostril and mononostril techniques by Wen et al. [60]. This meta-analysis involving 4805 patients compared binostril and mononostril surgical approaches for macroadenomas, revealing similar rates of gross total resection, hormonal remission, and complications between the two. The binostril group had lower rates of temporary diabetes insipidus and anterior pituitary insufficiency, as well as shorter hospital stays, while the mononostril group experienced less epistaxis. Additionally, the binostril approach suggested a tendency for better outcomes in invasive macroadenomas, although no subgroup analysis was performed [60]. Overall, there are many debates of using binostril or mononostril approaches for endonasal skull base surgery biased by authors preference and institutional setting. There is a lack of randomized or comparative multicenter trials to date to compare these surgical techniques. Therefore, the authors are only able to demonstrate small comparative series (see Table 5) and describe their results to their findings.

### Study Limitations

The authors acknowledge that the variability in the follow-up evaluation time period in the analyzed series may affect the generalizability of the results, particularly concerning long-term functional outcomes such as hormonal preservation and pituitary insufficiency. Additionally, a potential bias of selection may have impacted the study results, and the authors’ preference for a mononostril approach could introduce procedural bias, limiting the applicability of the findings to other surgical techniques. Addressing these limitations is essential for a comprehensive understanding of this study’s implications and for guiding future research.

## 5. Conclusions

In conclusion, the presented data suggests that the choice between a mononostril and binostril approach does not significantly affect visualization during endonasal surgery. The use of angled scopes in a mononostril approach allows for gross total tumor resection, offering adequate operating space and instrument maneuverability after a relatively brief learning curve. Nevertheless, the limitations and difficulties of the mononostril approach should be taken into account. The authors advocate for the binostril technique when addressing transclival approaches or tumors that necessitate larger corridors for improved maneuverability, e.g., behind the internal carotid artery or in the cavernous sinus. To further assess the efficacy of the mononostril approach, additional studies are required that provide detailed technical insights, long-term postoperative results, and randomized multicentric trials comparing the two techniques.

## Figures and Tables

**Table 1:** Characteristics and pathologies analyzed in the presented mononostril series in the literature.

	Jho *n* = 50	Cappabianca *n* = 146	Linsler *n* = 218	Han *n* = 200	Eseonu *n* = 275	Darwish *n* = 64	Oertel *n* = 55	Peeters *n* = 512
**Mean age**	38 years (14–88 yrs)	46.06 years (16–74 yrs)	58 years (23–83 yrs)	43.8 years (19–71 yrs)	50.4 years (18–85)	40 yrs	55.3 yrs	48.1 yrs
**Mean follow-up evaluation period**	6 months	6 months	4.61 yrs	4–42 months	6 months	4–72 months	6.2 yrs	1–10 yrs
**Pathologies:** Non-functioning adenomas	38%	55%	68%	60.5%	63.5%	39%	58%	62.6%
GH-secreting adenomas	0	25%	13%	16.5%	17.2%	18%	0	16.4%
Prolactinomas	34%	9.5%	4%	12.5%	2.8%	17%	0	8%
ACTH-secreting adenomas	16%	9.5%	2.5%	8.5%	15.3%	26%	0	12.5%
TSH-secreting adenomas	0	1%	0	1%	0.75%	0	0	0.5%
Craniopharyngioma	2%	0	0	0	0	0	9%	0
Clivus chordoma	2%	0	1.5%	0	0	0	7%	0
Rathke’s cleft cyst	2%	0	5%	0	0	0	6%	0
Meningiomas	0	0	0	0	0	0	11%	0
Others	6%	0	6%	0	0	0	9%	0

**Table 2:** Comparison of the radicality and recurrence rate of the analyzed studies (n.a. = not available).

Radicality	Jho *n* = 50	Cappabianca *n* = 146	Linsler *n* = 218	Han *n* = 200	Eseonu *n* = 275	Darwish *n* = 64	Oertel *n* = 55	Peeters *n* = 512
All non-functioning adenomas	84%	56%	88%	-	85.1%	74%	93%	-
Non-invasive adenomas	100%	-	91%	94%	-	78%	-	87%
Invasive and giant adenomas	0/3	-	0%	0%	-	13%	-	75%
Prolactinomas		77%	89%	87.5%	85%	n.a.	-	
ACTH adenoma	75%	77%	84%	81%	87.9%	82%	-	81%
GH adenoma	-	64%	85%	72%	87.9%	54%	-	58%
Rathke’s cleft cyst	100%	-	83%	-	-	-	100%	-
Meningiomas	-	-	-	-	-	-	83%	-
Craniopharyngiomas	-	-	-	-	-	-	100%	-
Others	-	-	-	-	-	-	100%	-
**Recurrence rate**	n.a.	n.a.	2.2%	n.a.	n.a.	n.a.	4%	8.2%

**Table 3:** Complications reported in the identified studies (n.a. = not available).

	Jho *n* = 50	Cappabianca *n* = 146	Linsler *n* = 218	Han *n* = 200	Eseonu *n* = 275	Darwish *n* = 64	Oertel *n* = 55	Peeters *n* = 512
Sellar hematoma	0	1%	0.5%	n.a.	n.a.	n.a.	4%	0
CSF fistula	4%	2%	3.2%	3.5%	3.6%	1.4%	2%	4.1%
Meningitis	0	1%	1.4%	0.5%	1%	1.4%	2%	n.a.
Worsening of visual deficit	0	1%	0.5%	0	5.6%	0	2%	2%
Brain infarction	0	0	0	0	0.7%	0	0%	0
ICA injury	0	1%	0	0.5%	0.4%	0	0	0
CN VI palsy	0	1%	0	0	0	0	0	0
Sinusitis	0	2%	0	6%	0.4%	n.a.	0	n.a.
Epistaxis	n.a.	1.4%	0	6%	0.4%	1.4%	0	n.a.
Cacosmia	n.a.	1.4%	1.2%	6%	0.4%	0	0	2.1%
Diabetes insipidus	n.a.	n.a.	0.4%	5%	0.7%	1.4%	3%	3.7%
Hormonal dysfunction	n.a	n.a.	n.a.	3%	4.8%	9.3%	4%	n.a.
Death	n.a.	0.7%	0	0.5%	0	0	0	0

**Table 4:** Differences between the surgical techniques described in the analyzed series (n.a. = not available).

	Jho *n* = 50	Cappabianca *n* = 146	Linsler *n* = 218	Han *n* = 200	Eseonu *n* = 275	Darwish *n* = 64	Oertel *n* = 55	Peeters *n* = 512
Speculum	No	No	Yes	Yes	No	No	Yes	No/yes
Endoscope holding device	Yes	No	Yes	No	Yes	No	Yes	Yes
2-hand technique	Yes	No	Yes	No	Yes	No	Yes	Yes
3-/4-hand technique	No	Yes	No	Yes	No	Yes	No	No
Neuronavigation	No	Yes	Yes	Yes	Yes	Yes	Yes	Yes
Lateral fluoroscopy	Yes	Yes	Yes	No	No	No	Yes	Yes
Mean surgical time	n.a.	n.a.	87 min	115 min	161.6 min	168 min	90.1 min	118 min

**Table 5:** Comparison of binostril and mononostril approach. (**A**) Comparison by Han et al. [39] (**B**) Comparison by Conrad et al. [25] (n.s. = not significant).

*n* = 250	Binostril *n* = 50	Mononostril *n* = 200	Significance
**(A)**			
Gross total resection rate	84%	87%	n.s.
Hormonal remission	79%	81%	n.s.
Visual improvement after surgery	87.5%	91%	n.s.
CSF leak	4%	3.5%	n.s.
Diabetes insipidus	2%	4.5%	n.s.
Pituitary insufficiency	2%	2.5%	n.s.
Surgical time (min)	156 ± 17.3	115 ± 15.2	s.
Blood loss	150 ± 18.3	94 ± 20.5	s.
Recurrence rate	None	None	
Progression	None	None	
**(B)**			
surgical time (min)	123 ± 40	93 ± 28	n.s.
CSF fistula intraoperatively	6	4	n.s.
Restricting by nasal speculum	0	5	n.s.
Nasal packing required	0	4	n.s.
Resolution of preoperative pituitary insufficiency	1/10	1/9	n.s.
New postoperative pituitary insufficiency	3	1	n.s.
ACTH deficiency	3	1	n.s.
Resolution of visual field deficits	7/8	5/6	n.s.
Residual tumor	4	2	n.s.
Diabetes insipidus	1	0	n.s.

## Data Availability

The original contributions presented in this study are included in the article. Further inquiries can be directed to the corresponding author.

## Supplementary Materials

The following supporting information can be downloaded at https://www.mdpi.com/article/10.3390/life15081233/s1, Table S1: PRIMSA2020 checklist [48].

## Abbreviations

The following abbreviations are used in this manuscript:

MRI	Magnetic resonance imaging
CT	Computer tomography
CSF	Cerebrospinal fluid
ICA	Internal carotid artery
ACTH	Adrenocorticotropic hormone
GH	Growth hormone
HD	High definition
PRIMSA	Preferred Reporting Items for Systematic Reviews and Meta-Analyses
